# MACHINE LEARNING APPLIED TO MEDICARE CLAIMS DATA TO IDENTIFY OLDER ADULTS WITH UNDIAGNOSED DEMENTIA

**DOI:** 10.1093/geroni/igae098.2276

**Published:** 2024-12-31

**Authors:** Deborah Barnes, Cynthia Benjamin, Delia Mannen, John Boscardin

**Affiliations:** University of California San Francisco, San Francisco, California, United States; Together Senior Health, San Francisco, California, United States; Together Senior Health, Cortland, Ohio, United States; University of California San Francisco, San Francisco, California, United States

## Abstract

Approximately half of people living with Alzheimer’s disease and related dementias are undiagnosed. We performed a retrospective cohort study using the 5% Medicare claims sample to test machine learning approaches for calculating risk of undiagnosed dementia. For each calendar year from 2012 to 2021, we created a cohort of beneficiaries aged 65 years or older on January 1 who had at least 2 healthcare claims during the previous 2 years. Those who had prevalent dementia, were in hospice, or died within the calendar year were excluded. We identified those who received an incident dementia diagnosis during the calendar year (i.e., had undiagnosed dementia on January 1) and used machine learning methods to identify key predictors of undiagnosed dementia within each cohort based on demographics, diagnoses, and healthcare utilization. Model accuracy was assessed using c statistics. On average, 837,440 Medicare beneficiaries were included in each cohort, of whom 24,410 (2.9%) were diagnosed with incident dementia each year. An average of 35±10 variables were included using different machine learning approaches and cohort years. Overall model accuracy for detecting undiagnosed dementia ranged from 0.77 to 0.81. All models yielded clinically meaningful discrimination ranging from < 1% (lowest predicted decile) to 13% (highest predicted decile). Model performance was stable temporally and across gender, race, and ethnicity. These results support application of machine learning with Medicare claims to identify older adults at risk of undiagnosed dementia. Future studies should prospectively validate this approach within healthcare systems and explore the potential value of additional data sources.

